# A routine outcome measure for youth mental health: Clinically interpreting MyLifeTracker

**DOI:** 10.1111/eip.13016

**Published:** 2020-07-14

**Authors:** Benjamin Kwan, Debra J. Rickwood

**Affiliations:** ^1^ Faculty of Health University of Canberra Bruce Australian Capital Territory Australia; ^2^ headspace National Youth Mental Health Foundation Melbourne Victoria Australia

**Keywords:** clinically significant change, expected change, MyLifeTracker, routine outcome measure, youth mental health

## Abstract

**Aim:**

MyLifeTracker is a session‐by‐session mental health outcome measure for young people aged 12 to 25 years. The aim of this study was to determine clinically significant change indexes for this measure that would identify developmentally appropriate thresholds. The study also aimed to determine expected change trajectories to enable clinicians to compare a client's progress against average rates of change.

**Methods:**

Participants comprised young people aged 12 to 25 years from both a clinical and a community sample from Australia. The clinical sample was 63 840 young people that attended a *headspace* centre. The non‐clinical group was an Australian representative community sample of 4034 young people.

**Results:**

Clinically significant change indexes were developed for MyLifeTracker specific for age and gender groups by comparing clinical and non‐clinical samples. Males and young people aged 12 to 14 years needed to reach higher scores to achieve clinically significant change compared to females and other age groups, respectively. MyLifeTracker expected change trajectories followed a cubic pattern for those with lower baseline scores of 0 to 50, whereas those with baseline scores of 51 and above had varying patterns. For those with lower baseline scores, expected change trajectories showed that stronger change was evident early in treatment, which then tapered off before accelerating again later in treatment.

**Conclusions:**

The development of MyLifeTracker benchmarks allows the measure to be used for Feedback Informed Treatment by supporting treatment planning and decision‐making. This information can help clinicians to identify clients who are not on track or deteriorating and identify when clients are improving.

## 1 INTRODUCTION

MyLifeTracker (MLT) is a recently developed mental health outcome measure for routine monitoring specifically targeted for young people aged 12 to 25 years. It was co‐designed with both young people and youth mental health clinicians to assess meaningful outcomes in the domains of general wellbeing, daily functioning, relationships with friends, relationships with family and coping. MLT has shown evidence of a single factor structure, although the five items were also designed to be clinically useful individually. Overall, MLT measures the quality of life, with higher scores indicating higher levels of quality of life. It has been validated against measures of psychological distress, quality of life and wellbeing, and demonstrates appropriate reliability and sensitivity to change (Kwan, Rickwood, & Telford, 2018). MLT is currently implemented into an electronic data system used by the 110 *headspace* youth mental health services implemented across Australia (Rickwood et al., 2018). This electronic data system provides information to clinicians that are collected from clients prior to every visit and displayed to the clinician in the form of a graph over time of MLT scores. This reveals change over time that can be used by the clinician to ascertain treatment progress and can also be shown to clients during their session via a computer or tablet device.

MLT was developed to fill a measurement gap in youth mental health. Historically, outcome measures have been designed that reflect the traditional mental health service demarcation between the child and adolescent services, for those aged less than 18 years, and adult services, for those aged 18 years and above (Kwan & Rickwood, 2015). The growing implementation of youth mental health services internationally, which span the age range of 12 to 25 years, necessitates new measures (McGorry, Bates, & Birchwood, 2013). MLT was designed to be suitable for session‐by‐session use by being very brief and comprising only five items. An essential next step for the development of MLT is the identification of age and gender norms, which improves the interpretability of such measures (Centofanti et al., 2018). This information is particularly important in youth mental health because adolescence and early adulthood are periods of rapid social, emotional and physical development when age and gender differences are pronounced (Donald, Rickwood, & Carey, 2014; Rickwood et al., 2015).

The development of session‐by‐session measures for routine outcome monitoring supports Feedback‐Informed Treatment (FIT) approaches, whereby a clinician receives quantitative feedback on a client's progress to use in‐session and help guide treatment planning (Bickman, 2008). FIT requires a measurement system that is easily completed by the client and allows instant feedback to the clinician (Hall et al., 2014). This provides clinicians with regular up‐to‐date snapshots of a client's mental health status and shows any changes since past sessions (Lutz, De Jong, & Rubel, 2015). Clinicians are then able to monitor if clients are progressing or deteriorating between sessions, and adjust treatment planning accordingly (Boswell, Kraus, Miller, & Lambert, 2015). Such an approach can also allow clinicians to bring the measures into sessions and feedback progress to clients, which can be a powerful way to promote shared decision making (Reese, Norsworthy, & Rowlands, 2009). FIT has been shown to improve communication between client and clinician, increase the accuracy of diagnosis, enable quicker adjustments to treatment planning when required, provide stronger outcome effects and improve the efficiency of treatment (Bickman, Kelley, Breda, de Andrade, & Riemer, 2011; Carlier et al., 2012; Janse, De Jong, Van Dijk, Hutschemaekers, & Verbraak, 2017).

A valuable metric for clinicians to use in FIT is change scores, such as clinically significant change indexes, expected change trajectories and early warning signals. These can be calculated from session‐by‐session measures to provide evidence‐based benchmarks for FIT and routine outcome monitoring systems. A clinically significant change is conceptualized as the process of a client starting treatment in the dysfunctional (clinical) population and leaving treatment no longer in this population (Jacobson, Follette, & Revenstorf, 1984). It is operationalized as a change in a client's outcome measure score showing that they are statistically more likely to be drawn from the functional distribution, having moved out of the dysfunctional distribution during treatment (Jacobson & Truax, 1991). When the dysfunctional and functional populations are identified, clinically significant change indexes can be calculated by finding the value where the two populations intersect. Reliable change can also be determined, which takes into account the reliability of the outcome measure, ensuring that change is not due to measurement error. Change can be then categorized into four stages: Deterioration―when a client has reliably worsened; Unchanged―when no reliable change has occurred; Improvement―when a client has made a reliable positive change but still remains in the dysfunctional population and Recovered―when a client reliably improves and moves into the functional population (Jacobson & Truax, 1991).

A criticism of clinically significant change is that it can be an overly stringent measure of change, being based on diagnostic cut‐offs. In naturalistic clinical settings, some clients may not be able to reach this threshold because they initially present in the functional population range (Wise, 2004). Other methods of monitoring change have been recommended; specifically, the use of growth curve modelling, which shows expected rates of change (Donald & Carey, 2017). This approach estimates a mean starting point (intercept) and average rates of change (slope) of the pooled sample trajectory; that is, within‐person expected change patterns (Singer & Willett, 2003). The method is particularly useful for exploring client change in naturalistic therapy settings as it can deal with data that are time‐unstructured and unbalanced. This provides clinicians with an expected change trajectory, which can be compared with an individual client's trajectory to determine whether the client is within or outside expected rates of change, potentially indicating the cause for concern (Finch, Lambert, & Schaalje, 2001).

Research has increasingly focussed on detecting clients who are at risk of deterioration using early warning systems that are derived from expected change trajectories (Finch et al., 2001). An early warning is evident when a client's score drops below an identified threshold. It is recommended that these early warning signals be derived from the bottom‐end percentage of the targeted population and the proportion of clients who reliably deteriorate in that population (Finch et al., 2001; Warren, Nelson, Mondragon, Baldwin, & Burlingame, 2010). An essential aspect of early warning signals is the ability to accurately predict clients who are responding poorly to treatment or are not on track(NOT) before therapy is terminated(Boswell et al., 2015). Some studies have evaluated the efficacy of these signals of deterioration, alerting clinicians to clients that are falling into the bottom 10% to 20%, demonstrating detection accuracy rates of 85% to 100% when used with adult clients (Lambert et al., 2002). Lower detection accuracy rates of 69% to 88% are seen when early warning signals are used with children and adolescents, which has been justified by the higher proportions of treatment failure when compared to adult clients (Cannon, Warren, Nelson, & Burlingame, 2010; Nelson, Warren, Gleave, & Burlingame, 2013; Warren, Nelson, & Burlingame, 2009).

Therapeutic deterioration is evident in up to 10% of adult clients (Lilienfeld, 2007; Murphy, Rashleigh, & Timulak, 2012), but much higher at 21%, for clients in youth psychotherapy settings (Warren et al., 2009). High dropout rates are another major concern in youth mental health settings, and dropout has been shown to be partly due to clinician and therapeutic factors that may be responsive to feedback (de Haan, Boon, de Jong, Hoeve, & Vermeiren, 2013). Early warning alerts have been shown to reduce deterioration from 20.1% in treatment as usual to 5.5% in feedback conditions for adult clients (Shimokawa, Lambert, & Smart, 2010). Feedback was also shown to double the proportion of clients with clinically significant improvement in NOT clients. Feedback to clinicians alone, and to both clinician and client, has been shown to significantly positively increase the rates of change in short‐term adult NOT clients (De Jong et al., 2014).

FIT approaches are increasingly being advocated because clinicians have been shown to have low accuracy rates of predicting client deterioration during therapy when using their judgement alone (Hannan et al., 2005; Hatfield, McCullough, Frantz, & Krieger, 2010). It is proposed that clinicians have a self‐assessment bias which serves to maintain a positive self‐image (Parker & Waller, 2015). For example, Walfish, McAlister, O'Donnell, and Lambert (2012) explored clinicians' ratings of their own clinical skills and client outcomes, showing that they rated their skills on average at the 80th percentile and that all clinicians rated themselves above the 50th percentile. In addition, clinicians on average believed that 77% of their clients improved as a result of their therapeutic intervention, which is well above the one‐third proportion of clients shown to improve in most naturalistic settings (Walfish et al., 2012). Deliberate practice, incorporating FIT with evidence‐based benchmarks, could be very effective at reducing this self‐assessment bias amongst clinicians (Chow et al., 2015; Goodyear, Wampold, Tracey, & Lichtenberg, 2017; Macdonald & Mellor‐Clark, 2015). Despite the potential clinical utility, however, clinicians have been shown to have limited knowledge around the use of routine outcome measures in predicting client deterioration (Bystedt, Rozental, Andersson, Boettcher, & Carlbring, 2014).

The current study investigated the implementation of routine outcome measurement and clinician feedback within youth mental health services, using the MLT measure. We aimed to determine MLT clinically significant change indexes that would identify developmentally appropriate thresholds for different age and gender groups. It was anticipated that there would be different clinically significant change indexes across the developmental period between 12 and 25 years and between males and females, due to the major changes that take place during adolescence and early adulthood and the marked gender differences in mental health status between males and females (eg, females displaying higher levels of psychological distress) (Brann, Lethbridge, & Mildred, 2018; Centofanti et al., 2018; Kwan et al., 2018). Identifying these developmental patterns would allow clinicians to provide more tailored client care. To do this, scores for a clinical population group were compared with data from a nationally representative community sample to determine appropriate change indexes. It was hypothesized that the non‐clinical group would have higher MLT scores compared with the clinical group, that males would have higher MLT scores than females, and that the younger adolescents would have higher MLT scores than those who were older (Kwan et al., 2018). We also aimed to determine expected change trajectories and early warning signals for MLT to provide benchmarks to help clinicians identify if a client is showing expected change over time in treatment, or whether the client is deteriorating. Lastly, we provide examples of how clinicians can use the statistically derived benchmarks for MLT in their clinical practice.

## 2 METHODS

### 2.1 Participants

Participants comprised both a clinical and a nationally representative community sample. The clinical sample was 63 840 adolescents and young adults between the age of 12 and 25 years who commenced the first episode of care at a *headspace* centre. *headspace* is the Australian Government's National Youth Mental Health Foundation, which was initiated in 2006 to provide early intervention in youth mental health. *headspace* centres offer services responding to mental health, alcohol and other drugs, general health and vocational concerns for young people (Rickwood et al., 2015).This sample consisted of 40.4% males and 59.6% females, in the following age ranges: 12 to 14 years (24.1%), 15 to 17 years (32.0%), 18 to 21 years (29.1%) and 22 to 25 years (14.8%).

The non‐clinical group was a nationally representative community sample that consisted of 4034 young people aged 12 to 25 years from across Australia. The sampling was stratified to provide a near‐even split between males (49.1%) and females (50.9%), and across age groups: 12 to 14 years (24.7%), 15 to 17 years (24.7%), 18 to 21 years (25.0%) and 22 to 25 years (25.6%).

### 2.2 Procedure

The clinical group commenced their first episode of care at a *headspace* centre between July 1, 2015 and March 31, 2017. During this period, data were available for 101 *headspace* centres across Australia. *headspace* centres routinely collect a minimum dataset comprising data from young people and their service providers at every occasion of service. The dataset includes demographic characteristics, clinical presentation and treatment outcome measures. Young people can present for a wide range of reasons to *headspace* centres (Rickwood et al., 2015), but only those who were deemed by their clinician to be at one of the following stages of mental illness were included in the current analyses: mild to moderate general symptoms; sub‐threshold diagnosis; threshold diagnosis; periods of remission or ongoing severe symptoms.

The data from *headspace* centres are encrypted and uploaded to a national datawarehouse, which is used for research, monitoring and evaluation. Ethics approval was obtained through quality assurance processes, comprising initial consideration and approval through the *headspace* board research sub‐committee. The consent processes have been reviewed and endorsed by an independent body, the Australasian Human Research Ethics Consultancy Services.

The non‐clinical group was recruited between July and September 2018. A research consultancy agency was commissioned by *headspace* to undertake a national computer‐assisted telephone interview of young people aged 12 to 25 years from across Australia. A quota sampling procedure was used to ensure equal numbers by gender and age group. The sample was recruited through random digit dialling (RDD; randomly generating Australian mobile phone and landline numbers). Ethics approval was obtained from Bellberry Limited Human Research Ethics Committee.

### 2.3 Measures

Both the *headspace* minimum dataset and *headspace* nationally representative community survey include a large number of demographic, clinical and outcome measures. For the current study, only the demographic characteristics of gender (male, female, other), age group(12‐14, 15‐17, 18‐21 and 22‐25 years), and the MLT routine outcome monitoring measure were used.

#### 2.3.1 Routine outcome monitoring measure

MLT (Kwan et al., 2018) is a five‐item self‐report measure used to assess the current quality of life in areas of importance to young people. It asks young people to indicate how they have been feeling over the last week in relation to their: “general wellbeing (emotional, physical, spiritual)”, “day‐to‐day activities (study, work, leisure, self‐care)”, “relationships with friends”, “relationships with family” and “coping (dealing with life, using your strengths)”. Responses are given on a sliding scale anchored at 0 and 100 with the chosen score visible, accompanied by a visual analogue of a sad and happy face as anchors. Total MLT scores were calculated by averaging across the five items, ranging from 0 to 100, with a higher score indicating a higher quality of life. In the present study, internal consistency was high, with the Cronbach's *α* = .83 in the clinical group and .88 in the non‐clinical group. The original MLT study reported a Cronbach's *α* of .84, which ranged from .79 to .86 across age groups and gender (Kwan et al., 2018).

### 2.4 Data analyses

SPSS V21 was used for all analyses. First, descriptive statistics for MLT were calculated and a factorial between groups analysis of variance (ANOVA) was conducted to evaluate the differences in MLT scores across population groups (clinical, non‐clinical), gender (male, female) and age groups (12‐14, 15‐17, 18‐21, 22‐25 years). Games‐Howell post‐hoc tests were conducted to address unequal variances and sample sizes. Due to the large sample size, a significant change was reported as partial *η* ^2^ > .001 and *d* ≥ .02.

Clinically significant change indexes were calculated using data from the clinical and non‐clinical samples for each age group and gender (male and female; there were too few participants reporting non‐binary gender in the non‐clinical sample to create a third gender group) combinations. Results from the original MLT study revealed differences in baseline MLT scores across age and gender groups (Kwan et al., 2018). The formula proposed by Jacobson and Truax (1991) was used to calculate clinically significant change indexes when both clinical and non‐clinical groups are available but have unequal variances (p. 13).

Expected change trajectories were determined for the clinical group using growth curve modelling (Singer & Willett, 2003), which estimated average rates of change in MLT composite scores across participants' episodes of care. This approach was utilized as it provides fixed effects that estimate a mean slope of the pooled sample trajectory (within‐person patterns). Maximum likelihood estimation procedures were used. Weeks in treatment were used over session number as the time variable because this has been recommended in the past literature exploring youth psychotherapy change (Warren et al., 2010) and provided a better model fit based on Bayesian Information Criterion (BIC).

Expected change trajectories were calculated for decile groups dependent on MLT baseline scores; that is, 0 to 10, 11 to 20, etc. A precedence has been set for this method by past research exploring change trajectories, which show differing rates of change dependent on baseline severity on outcome measures (Finch et al., 2001; Lambert et al., 2002). Only data from participants attending more than one session and with treatment length up to 26 weeks were used to avoid extreme outliers in terms of treatment length. Two early warning signals were calculated based on the baseline MLT score and expected change trajectory: one SD below the expected change trajectory and reliable deterioration based on the baseline MLT score.

## 3 RESULTS

### 3.1 Clinically significant change indexes

Table 1 provides the descriptives for MLT scores for the clinical and non‐clinical groups, and the calculated clinically significant change indexes for MLT across age groups and gender. The ANOVA revealed no significant interactions (partial *η* ^2^ ≤ .001) and only significant main effects. MLT scores were significantly higher in the non‐clinical group compared to the clinical group (partial *η* ^2^ = .149); and for males compared with females (partial *η* ^2^ = .005). MLT scores differed significantly by age group (partial *η* ^2^ = .013), and post‐hoc analyses revealed that scores for those aged 12 to 14 years were significantly higher than all other age groups (15‐17 years (*d* = .25), 18‐21 years (*d* = .36) and 22‐25 years (*d* = .24)), which did not differ significantly from each other (*d* < .20).

**TABLE 1:** Descriptive statistics for MyLifeTracker for the clinical and non‐clinical groups, and clinically significant change indexes, by age group and gender

	12‐14 years M (SD)	15‐17 years M (SD)	18‐21 years M (SD)	22‐25 years M (SD)
Clinical group				
Males	(n = 5008) 60.22 (21.03)	(n = 6543) 51.44 (19.68)	(n = 6563) 45.02 (19.14)	(n = 3444) 43.53 (18.99)
Females	(n = 8189) 48.24 (20.00)	(n = 10 855) 42.97 (17.87)	(n = 8921) 40.62 (17.70)	(n = 4240) 41.25 (18.36)
Non‐clinical group				
Males	(n = 519) 84.63 (12.49)	(n = 470) 80.51 (13.20)	(n = 484) 75.86 (16.40)	(n = 494) 76.66 (15.55)
Females	(n = 465) 83.71 (15.31)	(n = 525) 73.61 (17.76)	(n = 516) 72.15 (16.03)	(n = 536) 74.87 (15.87)
Clinically significant change indexes				
Males	75.53	68.84	61.63	61.74
Females	68.33	58.34	57.17	59.28

Females showed a lower threshold to achieve clinically significant change when compared to males across all age groups. Within gender, for both male and female participants, those aged 18 to 21 years showed the lowest threshold for clinically significant change and those aged 12 to 14 years showed the highest threshold when compared to the other age groups. Across all gender and age group combinations, females aged 18 to 21 years, 15 to 17 years and 22 to 25 years showed the lowest thresholds for clinically significant change, in that order. Males aged 12 to 14 years and 15 to 17 years had the highest clinically significant change indexes across all age groups and gender combinations.

### 3.2 Expected change trajectories

**FIGURE 1:**
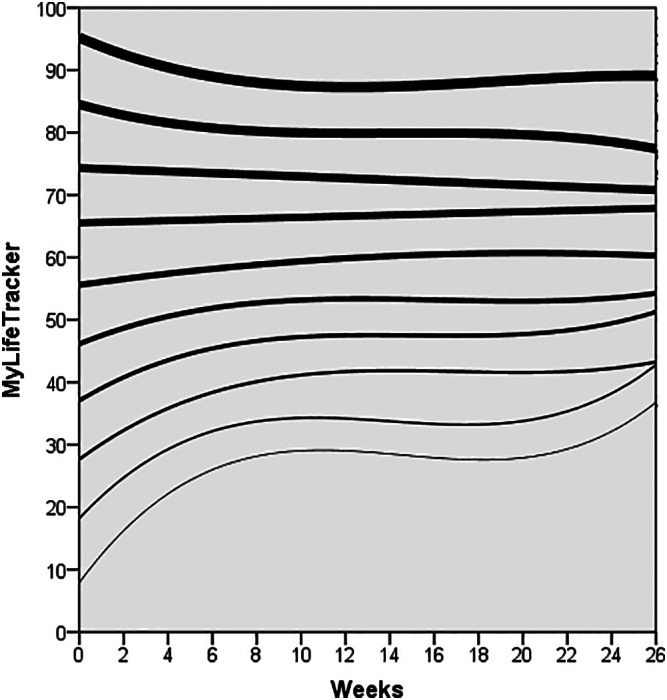
Expected change trajectories by baseline MyLifeTracker scores

Figure 1 presents the expected change trajectories by baseline MLT scores in deciles, and Table 2 shows the growth curve model slope estimates. The expected change trajectories followed a cubic pattern for those with a baseline score of 0 to 50; a quadratic pattern for baseline scores of 51 to 60; a linear pattern for baseline scores of 61 to 70; and non‐significant change over time for baseline scores of 71 to 80. MLT baseline scores of 81 to 100 again followed a cubic pattern; however, this was inverse to change trajectories seen in MLT baseline scores of 0 to 50. Within baseline scores between 0 and 50, expected change trajectories for the lower scores showed a steeper increase (linear growth), greater deceleration (quadratic growth) and a bigger acceleration (cubic growth) compared with higher scores. A similar trend was evident for MLT scores between 81 and 100, but in the opposite direction, trending downwards.

**TABLE 2:** Growth curve models for MyLifeTracker scores during treatment by baseline score

Baseline MLT score	Slope estimates (SE)		
	Linear (weeks)	Quadratic (weeks^2^)	Cubic (weeks^3^)
0‐10	5.04 (0.19)	−0.36 (0.02)	0.008 (0.0006)
11‐20	3.77 (0.11)	−0.26 (0.01)	0.006 (0.0004)
21‐30	2.82 (0.07)	−0.17 (0.008)	0.004 (0.0002)
31‐40	2.17 (0.05)	−0.13 (0.006)	0.003 (0.0002)
41‐50	1.31 (0.05)	−0.07 (0.005)	0.001 (0.0002)
51‐60	0.56 (0.03)	−0.006 (0.001)	NS
61‐70	0.27 (0.02)	NS	NS
71‐80	NS	NS	NS
81‐90	−1.16 (0.13)	0.10 (0.02)	−0.002 (0.0005)
91‐100	−1.83 (0.17)	0.14 (0.02)	−0.003 (0.0006)

*Note:* Slope estimates are growth curve model coefficients, Standard Error (SE), only significant estimates are shown.

### 3.3 Early warning signals for use in clinical practice

**FIGURE 2:**
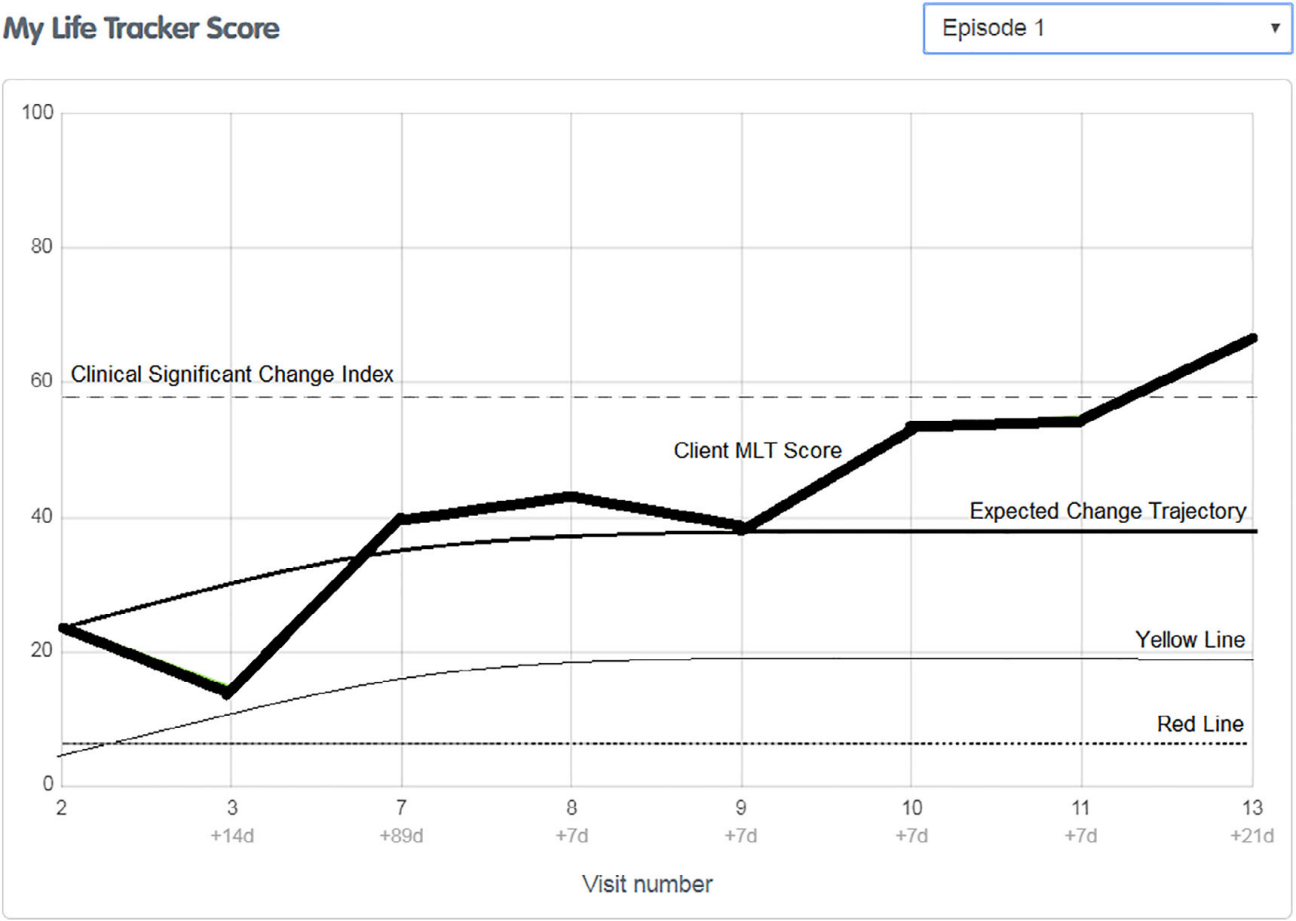
MyLifeTracker graph with benchmarks and early warning signals for a 15 to 17 year old female with a baseline score between 21 and 30

Two early warning signals were calculated: the first was a growth curve one SD below the expected change trajectory (SD = 19.81, the yellow line in Figures 1 and 2), which would warn that the client had fallen below the 16th percentile of expected change while in treatment. The yellow line would be relevant only for MLT baseline scores of 0 to 70 as they have an increasing trend, and MLT scores for 71 to 100 would not be necessary as they would reach reliable deterioration before they dropped below one SD of the expected change trajectory. The second early warning signal (red line in Figures 1 and 2) indicates when a client has reliably deteriorated from their baseline MLT score. Reliable change has previously been calculated for MLT to be a change of 18.27 points, and reliable deterioration would mean the client has dropped 18.27 points below their baseline score (Kwan et al., 2018). The red line would be relevant for all baseline MLT scores.

### 3.4 Clinical MLT examples

**FIGURE 3:**
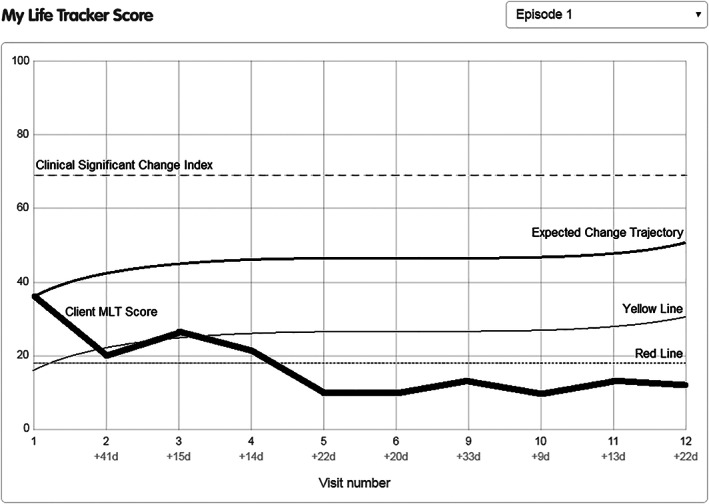
MyLifeTracker graph with benchmarks and early warning signals for a 12 to 14 year old female with a baseline score between 31 and 40

Figures 2 and 3 provide a visual graph of the type of information that could be provided to clinicians. Currently, in *headspace* centres, clinicians are provided only with graphs of MLT scores over time, but the inclusion of these newly calculated benchmarks would give additional clinically useful information to help interpret the MLT scores.

The first example in Figure 2 shows a positive therapeutic change directory. It is of a 15 to 17 year old female who presented with a baseline MLT score of 24. This would mean the young person would need to reach 58.34 on MLT to obtain clinically significant change. The expected change trajectory will start at an MLT score of 24 and follow the growth curve plotted for baseline MLT scores between 21 and 30. If her score drops below the yellow line she would be one SD (SD = 19.81) below the expected change trajectory or below the 16th percentile of expected change. If her MLT score further dropped equally to or below an MLT score of 5.73 during treatment (18.27 points below baseline MLT score), indicated by the red line, she would have reliably deteriorated. For this young person, her score drops to 15 in the second session, but this score is still above the yellow line, which means it is within one SD of expected change. By session seven, her MLT score is above the expected change trajectory for young people with baseline MLT scores of 21 to 30. Her progress remains above the expected change trajectory, which indicates she is making similar or better progress compared with other young people in treatment who started with a similar MLT score. At sessions 10 and 11, the young person's MLT score is still under the clinically significant change index but her score has increased above the 18.27 points (reliable change) from her baseline indicating reliable “improvement”. By session 13, she has an MLT score of 66, which is above the clinically significant change index, meaning that this young person has moved out of the clinical population. The change can be categorized as “recovered” as the young person has reliably improved and moved from the dysfunctional population into the functional range (Jacobson & Truax, 1991).

The second example, shown in Figure 3, shows a negative therapeutic change directory. It is of a 12 to 14 year old female with a baseline MLT score of 36.40. The clinically significant change index would be 68.33 and her expected change trajectory would follow that of clients with baseline MLT scores between 31 and 40. By session two, this young person has a score of 20, which alerts the clinician that she has dropped below the yellow line. In the third session, the young person has a score of 26, which brings her back above the yellow line, but by session four she dips back below the yellow line with a score of 21.40. In session five, the young person has an MLT score of 10.40, which indicates she has dropped below the red line and the young person remains below the red line for the remaining sessions. In this example, the first early warning signal (yellow line) is triggered at two‐time points, which tells the clinician that the client is dropping below one SD of expected change and that treatment planning may need to be reviewed. The second early warning signal (red line) is triggered by session five, showing the client has reliably deteriorated, and treatment planning and current support needed to be reviewed.

## 4 DISCUSSION

The current paper aimed to develop a set of clinically significant change indexes, expected change trajectories and early warning signals to help clinicians to interpret MLT for young people aged 12 to 25 accessing youth mental health services. Using comparative scores from a nationally representative non‐clinical sample, clinically significant change score benchmarks were able to be derived to assess client progress throughout treatment. Two examples were presented to demonstrate how the newly created benchmarks and early warning signals could be used to inform clinical practice. Table 3 summarizes the clinical benchmarks for MLT, which in conjunction with the other tables and figures, provides a quick guide of how clinicians can use and interpret MLT.

**TABLE 3:** Summary of clinical benchmarks for MyLifeTracker

Term	Definition
Clinically significant change index	This index provides clinicians with information on whether a client is more likely to be in the non‐clinical (above the index) or clinical population (below the index). It allows clinicians to see when a client moves from the dysfunctional to the functional population group during treatment, known as “clinically significant change”. These indexes are calculated by finding the value where the non‐clinical and clinical populations intersect. MyLifeTracker has clinically significant change indexes based on gender and age group (see Table 1). When a reliable change (18.27 points) is also considered, change can be categorized into four stages: Recovered―when a client reliably improves and moves into the functional population Improvement―when a client has made a reliable positive change but still remains in the dysfunctional population Unchanged―when no reliable change has occurred Deterioration―when a client has reliably worsened (see below in “Early warning signals—Red line” section) *Note:* If a client is above the clinically significant change index, a client cannot reach “recovered” and it may be difficult to achieve reliable “improvement” due to how high the client's score is and because they are already more likely to be in the functional population. The client can still show reliable “deterioration”.
Expected change trajectory	This trajectory provides clinicians with estimates of average rates of change for clients. An individual client's trajectory can be compared with the average trajectory to determine whether the client is within or outside expected rates of change. These trajectories are calculated using growth curve modelling based on a clinical group during an episode of care. MyLifeTracker has expected change trajectories calculated for decile groups dependent on MyLifeTracker baseline scores, that is, 0‐10, 11‐20, etc (see Table 2 and Figure 1).
Early warning signals—Yellow line	This yellow line provides clinicians with a warning when a client drops one SD below the expected change trajectory. This would mean that the client has fallen below the 16th percentile of expected change while in treatment and that treatment planning may need to be reviewed. These yellow lines are modelled on the same growth curve as the expected change trajectories, however, start from 19.81 points (one SD) below the client's MyLifeTracker baseline score (see examples in Figures 2 and 3). *Note:* The yellow line for MyLifeTracker is only relevant for baseline scores of 0‐70 as they have an increasing trend, and scores for 71‐100 are not necessary as they would reach reliable deterioration (red line) before they dropped below one SD of the expected change trajectory.
Early warning signals—Red line	This red line provides clinicians with a warning when a client has reliably deteriorated from their baseline score during their course of treatment. This may indicate that the client has increased risk or concerns, is not responding to treatment and may prematurely dropout from treatment. Clinicians should review treatment planning and check if additional supports are required. These red lines are calculated as18.27 points (reliable change) below the client's MyLifeTracker baseline score (see examples in Figures 2 and 3). *Note:* The red line would be relevant for all MyLifeTracker baseline scores. However, the red line will not exist when the MyLifeTracker baseline score is too low as a client's score cannot drop below 0 during treatment.

As hypothesized, clinically significant change indexes were distinctly different across age groups and gender, with male adolescents showing a higher threshold by seven to 10 MLT points compared to female adolescents. While young adult males also showed a higher threshold than young adult females, this gap was smaller (two to four MLT points). Overall, the largest difference between indexes was between the males aged 12 and 14 years and females aged 18 and 21 years, with a difference of 18 MLT points. This clearly demonstrates the need for gender and age‐specific clinically significant change indexes to provide appropriate benchmarks responsive to the distinct developmental variances occurring during this rapidly changing time of life (Donald, Carey, & Rickwood, 2018; McGorry, Goldstone, Parker, Rickwood, & Hickie, 2014).

The expected change trajectories followed a cubic pattern for MLT baseline scores below 50, and this pattern of change has been demonstrated in other naturalistic settings (Baldwin, Berkeljon, Atkins, Olsen, & Nielsen, 2009). It was shown that patterns of change were faster with lower MLT baseline scores, compared to higher MLT baseline scores below 50, and this pattern has been shown with other studies exploring youth outcomes (Cannon et al., 2010). These models of change using MLT are consistent with previous research that suggests there is a likelihood of more sudden change early in treatment and then a deceleration as treatment progresses (Baldwin et al., 2009; Gaynor et al., 2003; Tang, Luborsky, & Andrusyna, 2002). There is an increasing, but slower, rate of change among baseline MLT scores between 51 and 70, whereas baseline MLT scores of 71 to 80 showed no change over time. This can be explained by MLT scores being closer to the clinically significant change indexes, and it is expected that there will be less change over time as clients are already closer to the functional distribution. Baseline MLT scores of 81 to 100 showed an inverse cubic pattern to those baseline MLT scores under 50, with the MLT scores declining, and this ceiling effect is common across outcome measures for clients that rate their mental health very positively (Higginson & Carr, 2001).

The results of this study add to the growing research towards increasing the utility of youth mental health outcome measures to support FIT implementation (Centofanti et al., 2018; Kodet, Reese, Duncan, & Bohanske, 2019; Mayworm, Kelly, Duong, & Lyon, 2020). Young people are shown to have higher rates of deterioration and clinicians are shown to have lower rates of accurately predicting deterioration compared to adults in mental health treatment (Cannon et al., 2010; Warren et al., 2009). They are also more likely to show higher treatment dropout and missed appointments, and it has been suggested that this is due to their perceptions around the usefulness of professional help and stigma related to this (O'Brien, Fahmy, & Singh, 2009). This higher level of disengagement is particularly seen with young people who are males, Aboriginal or Torres Strait Islander, aged over 18 years and living in rural areas. However, a high number of those who discontinue from treatment are shown to reengage in the future, and those young people may need to engage multiple times to meet their mental health needs (Seidler et al.). As such, the use of MLT in FIT targets the developmental period spanning the 12 to 25 age range that may be quite responsive to this type of monitoring during treatment (Donald et al., 2018; Langer & Jensen‐Doss, 2018).

The clinically significant change indexes, expected change trajectories and early warning signals developed here provide important information to help youth mental health clinicians interpret changes in MLT scores. The functionality to include these indexes in the current *headspace* data collection system is not yet available, although sophisticated electronic measurement systems, tailored to clients' age and gender and baseline outcome scores, are becoming available. Such information can be very helpful for clinicians, to inform clinical practice and provide feedback to clients, and also clinicians' own deliberate practice. Deliberate practice, which is a process of systematic effort to improve performance with the guidance of a supervisor, ongoing feedback relative to essential skills, and refinement and repetition of practice (Goodyear et al., 2017), has been shown to contribute to differences between clinicians in client outcomes, with the most effective clinicians engaging in 2.8 times more deliberate practice than other clinicians (Chow et al., 2015).

There are still mixed views among clinicians using FIT, however, and this seems to affect its effectiveness (Lucock et al., 2015; Lutz et al., 2015). De Jong, Van Sluis, Nugter, Heiser, and Spinhoven (2012) showed clinicians who used the measurement feedback provided to them had improved outcomes for those clients NOT. Specifically, female clinicians and clinicians reporting higher commitment to using FIT at the start of treatment were more likely to use the feedback provided from the measure. Further, clinicians who were more likely to trust feedback from sources external to their own opinion (low internal feedback propensity), had clients with faster rates of change compared to clinicians with a high internal feedback propensity. Clinicians with a strong focus on achieving success (promotion focussed) were more likely to achieve better outcomes using feedback when compared to clinicians who focus on preventing failures (prevention focussed) (De Jong & De Goede, 2015). At a service level, clinics that showed a better implementation of feedback systems were more likely to have measures completed and outcomes viewed by clinicians, which in turn led to a more positive impact on client outcomes (Bickman et al., 2016). Training is increasingly available in the area of FIT and future research should target how to improve clinicians' acceptability of feedback monitoring systems and how to enhance its implementation and effectiveness(Law & Wolpert, 2014).

The results of the current study should be interpreted in light of its limitations. Notably, the clinically significant change indexes, expected change trajectories and early warning signals were created for an early intervention mental health service for young people aged 12 to 25 years in Australia. Further research is needed to determine whether the benchmarks would apply to young people attending specialist or tertiary services. The indexes were developed using a community sample from Australia, and it is unknown whether similar MLT scores would be found in other countries. Replication in other regions of the world focusing on the development of youth mental health systems, like Canada, Ireland, the Netherlands and California, is warranted (McGorry, Trethowan, & Rickwood, 2019). Furthermore, the current study only explored expected change trajectories dependent on baseline MLT scores, as past studies have shown that this accounts for a significant amount of variance in the rate of change(Lambert et al., 2002). However, it may be important also to create expected change trajectories for other predictors, such as the client's diagnosis and presenting issues. For example, a study on substance abuse treatment found that while baseline mental health measures were a significant predictor of rates of change, employment status and baseline craving levels were also significant predictors of rates of change (Crits‐Christoph et al., 2015).

In conclusion, the development of these MLT benchmarks is an important step to increase the clinical utility of the measure. MLT was originally developed to fill a gap in the availability of routine outcome measures for youth mental health services provided to adolescents and young adults. The availability of these benchmarks, including clinically significant change indexes and expected change trajectories, enhances the clinical utility and interpretability of the measure (Boswell et al., 2015; Donald & Carey, 2017). Providing benchmarks that are age group and gender specific is also critical for this age range when there is a substantial developmental change occurring in multiple domains. The clinical benefits of FIT are becoming more widely known and have become part of the agenda for the future progression of psychotherapy (Emmelkamp et al., 2014; Lutz et al., 2015). It is essential that such practices can be applied in youth mental health, where dropout and lack of clinical change are particularly problematic. The implementation of routine outcome measures, like MLT, and the use of benchmarks that enable clinicians to determine developmentally appropriate change directories that reveal recovery, improvement, lack of change or deterioration, is essential to supplement clinical judgement to improve clinical practice and outcomes in youth mental health settings.

## CONFLICT OF INTEREST

All authors are either employed by *headspace* National or a *headspace* centre. The authors report no other conflicts of interest in this work.

## Data Availability

The data are available only to the collaborating researchers with the headspace National Youth Mental Health Foundation. Some data may be available upon reasonable request, but not all due to relevant data protection laws.
